# Acute Pancreatitis Secondary to Duodenal Diverticulitis: A Case Report

**DOI:** 10.7759/cureus.103845

**Published:** 2026-02-18

**Authors:** Abeer Qasim, Ornela Thartori, Rayan Alataa, Elona Shehi

**Affiliations:** 1 Internal Medicine, BronxCare Health System, New York, USA; 2 Gastroenterology, BronxCare Health System, New York, USA

**Keywords:** acute pancreatitis, duodenal diverticulitis, duodenal diverticulum, idiopathic pancreatitis, mrcp

## Abstract

Acute pancreatitis is an inflammatory condition of the pancreas that can result in significant clinical complications. Determining the underlying etiology is essential, as management and recurrence risk depend on identifying the cause. When routine evaluation fails to reveal a clear etiology, less common causes should be considered. We present the case of a 55-year-old woman with acute pancreatitis secondary to duodenal diverticulitis. This rare condition can lead to pancreatitis through local inflammation or obstruction of the pancreatic duct. This case highlights the importance of advanced imaging in identifying uncommon etiologies of pancreatitis when initial diagnostic workup is unrevealing.

## Introduction

The most common etiologies of acute pancreatitis are gallstones and alcohol consumption. However, in a significant number of cases, the cause remains unclear after initial investigation, leading to a diagnosis of idiopathic pancreatitis. This diagnostic uncertainty presents a clinical challenge, as failure to identify the underlying cause can lead to recurrent episodes and increased morbidity. Rare causes of pancreatitis include hypertriglyceridemia, hypercalcemia, drug-induced pancreatitis, pancreatic divisum, primary pancreatic lymphomas, and duodenal diverticula. Duodenal diverticula, while often asymptomatic, are an important and frequently overlooked cause of pancreatitis. Their clinical significance was first documented by the French pathologist Chomel in 1710 [1].

Diverticula are outpouchings that can develop anywhere along the gastrointestinal tract, most commonly in the large intestine [2]. In the small intestine, they are less frequent, with the duodenum being the second-most common site after the jejunum [3]. Duodenal diverticula are rare in individuals under 40, and their prevalence increases with age. Estimating the true prevalence is difficult; they are identified in 1%-6% of upper gastrointestinal contrast studies, 12%-27% of endoscopic examinations, and 15%-22% of autopsy cases [4].

We present the case of a 55-year-old woman who was admitted with acute pancreatitis. After excluding all common causes, an MRI with magnetic resonance cholangiopancreatography (MRCP) was performed, which revealed duodenal diverticulitis as the cause of her pancreatitis.

## Case presentation

A 55-year-old female with a history of gastroesophageal reflux disease, erosive esophagitis, and hypothyroidism presented to the emergency department with a one-day history of epigastric and right upper quadrant abdominal pain. She denied nausea, vomiting, tobacco, alcohol, or recreational drug use, and had no history of over-the-counter medication or supplement intake. Her family history was notable for maternal gastric cancer, and her past surgical history included hysterectomy and cholecystectomy for benign indications. Her home medications included pantoprazole and levothyroxine. The etiology of her hypothyroidism was not documented in the available records.

On physical examination, she was afebrile and hemodynamically stable. Her abdomen was tender in the epigastric region and right upper quadrant, without guarding. Murphy's sign was negative. Laboratory evaluation on admission is detailed in Table 1. 

**Table 1:** Laboratory findings on admission

Laboratory Test	Value	Reference Range
Lipase	1,256 U/L	13-60 U/L
Triglycerides	120 mg/dL	<150 mg/dL
Calcium	9.5 mg/dL	8.6-10.3 mg/dL
Alanine Aminotransferase (ALT)	35 U/L	7-56 U/L
Aspartate Aminotransferase (AST)	40 U/L	10-40 U/L
Gamma-Glutamyl Transferase (GGT)	45 U/L	5-40 U/L

**Figure 1:**
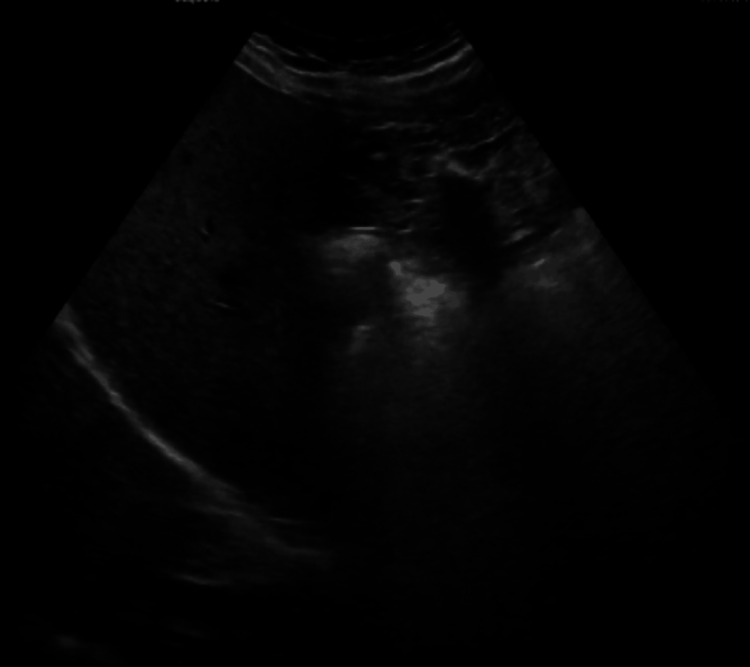
Abdominal ultrasound demonstrating post-surgical changes and no biliary obstruction

The diagnosis of acute pancreatitis was made based on the presence of two of the three required criteria: characteristic epigastric abdominal pain and elevated serum lipase to more than three times the upper limit of normal. The patient had a bedside index of severity in acute pancreatitis (BISAP) score of 1 on admission, indicating a low risk of mortality. Abdominal ultrasound showed a common bile duct of 0.5 cm, and the gallbladder was absent (prior cholecystectomy) (Figure 1).

**Figure 2:**
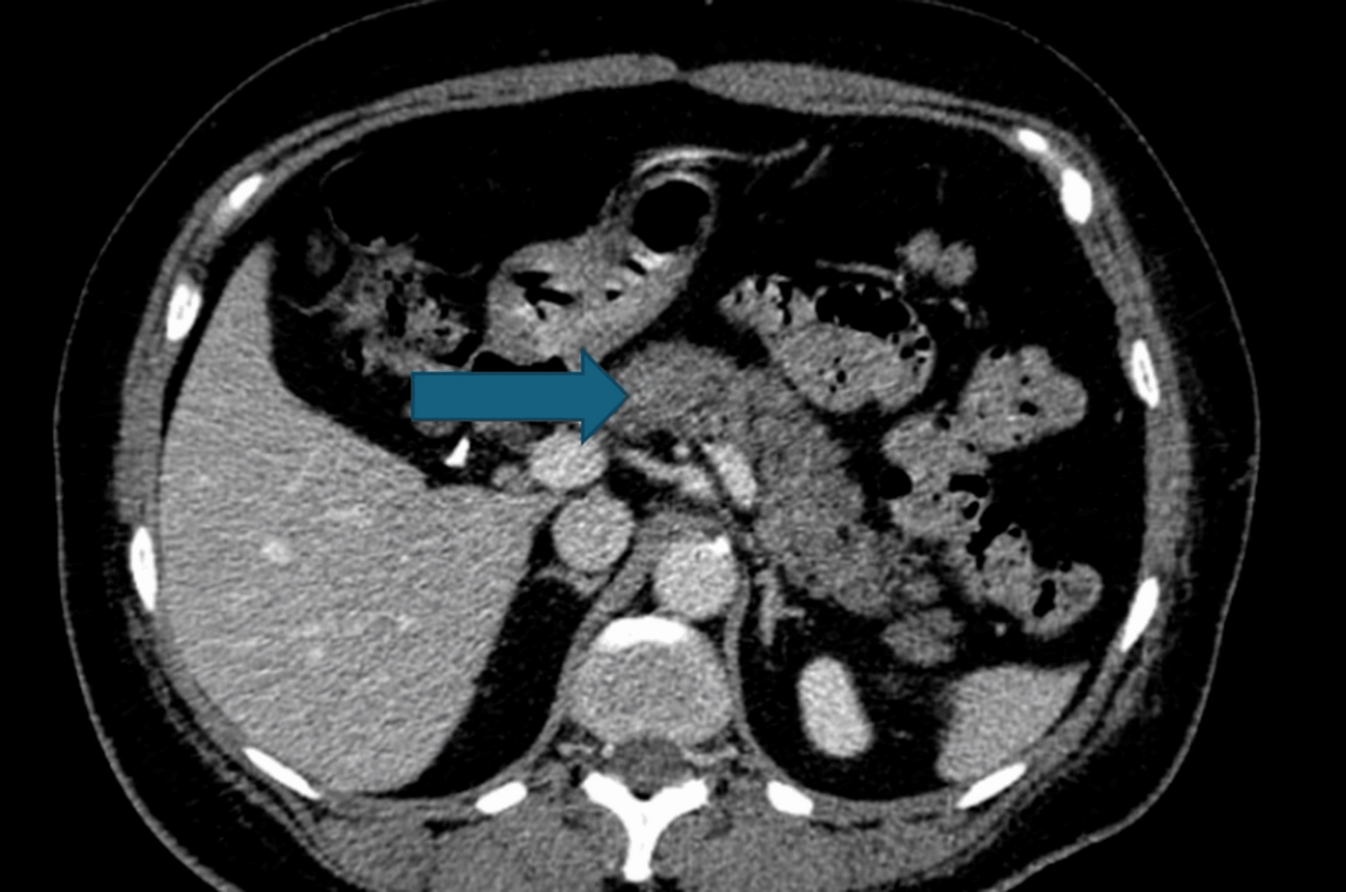
Acute pancreatitis on computed tomography An axial, contrast-enhanced computed tomography (CT) scan of the abdomen demonstrates inflammatory changes in the upper abdomen, characterized by peripancreatic fat stranding and soft tissue haziness concentrated around the head of the pancreas (indicated by the arrow). These findings are consistent with a diagnosis of acute interstitial pancreatitis.

She underwent a contrast-enhanced CT scan of the abdomen and pelvis, which revealed fatty deposition within the head of the pancreas with associated haziness of the interstitial fat planes of the pancreatic parenchyma, consistent with mild acute pancreatitis. Additional findings included evidence of prior cholecystectomy and hysterectomy, as well as diverticulosis (Figure 2).

**Figure 3:**
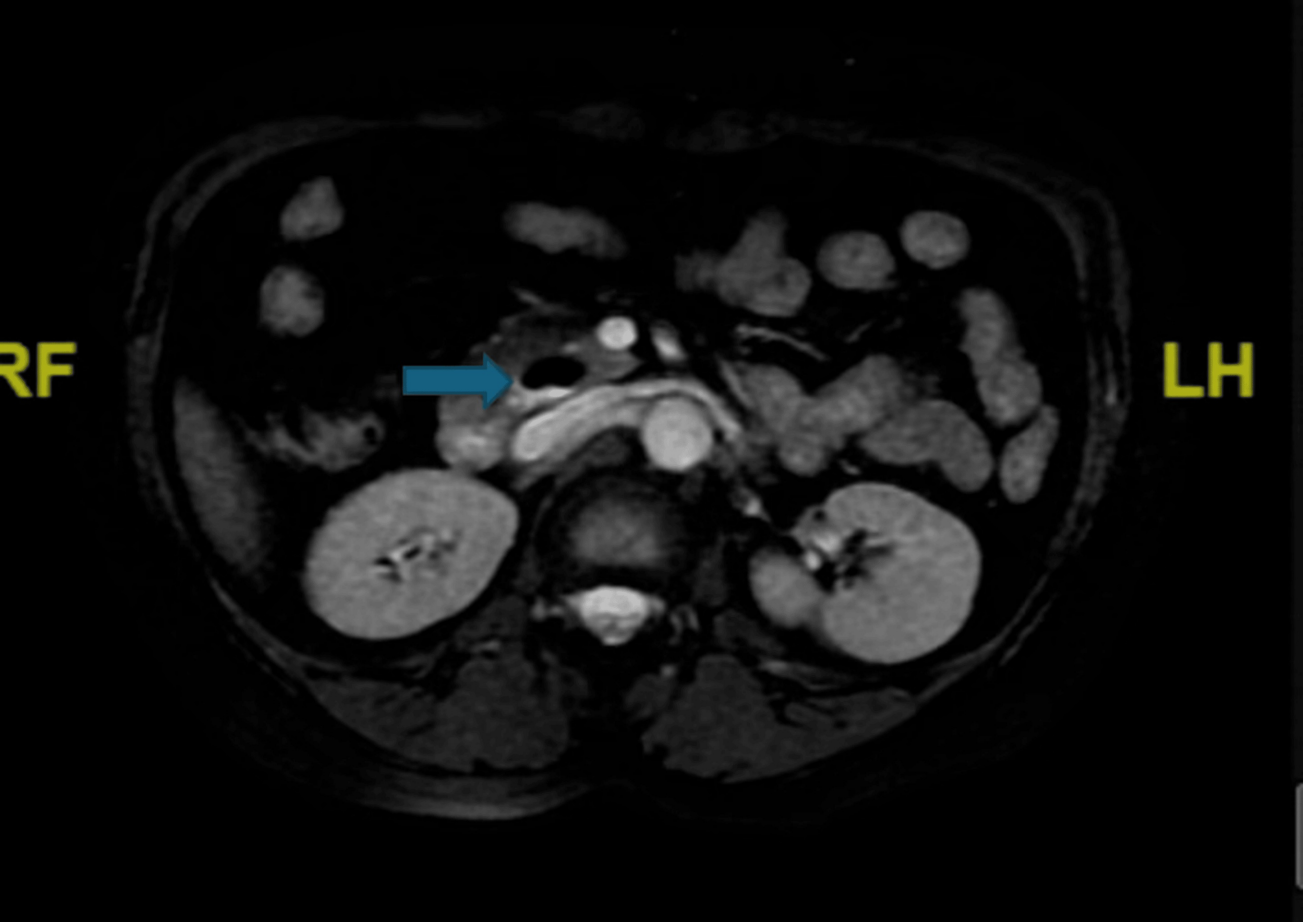
Duodenal diverticulitis identified on magnetic resonance imaging Axial T2-weighted magnetic resonance imaging (MRI) of the abdomen, performed as a follow-up study, reveals a small, well-circumscribed, fluid-filled outpouching arising from the second portion of the duodenum, consistent with a duodenal diverticulum (arrow). Surrounding high-signal intensity indicates associated inflammation (diverticulitis), establishing it as the definitive underlying cause of the patient's recurrent acute pancreatitis.

During her gastroenterology appointment, an MRI and MRCP were planned to rule out de novo stones and pancreatic malignancy as potential causes of her presentation. MRI of the abdomen demonstrated a small duodenal diverticulum near the head of the pancreas with surrounding haziness, suggestive of duodenal diverticulitis. MRCP revealed similar findings (Figure 3).

The patient’s condition improved without further intervention, and she remained well at her subsequent follow-up visit.

## Discussion

Duodenal diverticula are found in about 20% of the general population, with prevalence increasing with age [5]. The exact cause is unclear, but it may result from abnormal duodenal motility. Factors such as aging, weakening of the intestinal smooth muscle, and increased intraduodenal pressure contribute to their development [4]. Most duodenal diverticula do not cause symptoms; however, in some cases, complications drive the clinical presentation. Inadequate drainage or a narrow diverticular neck can promote inflammation, potentially resulting in bleeding or perforation.

Other complications occur in about 5% of cases, where biliopancreatic issues can arise due to direct compression of the common bile duct or sphincter of Oddi dysfunction [5]. Approximately 70-75% of duodenal diverticula are periampullary [6]. The link between periampullary diverticulitis and pancreatitis remains unclear; however, pancreatitis may result from mechanical compression or inflammation caused by the diverticulum, leading to narrowing of the duodenal papilla [7]. Several mechanisms have been proposed, including dysfunction of the sphincter of Oddi leading to reflux of pancreatic secretions and intestinal contents [8-10], and stasis within the diverticula contributing to both biliary and pancreatic complications [11]. In our patient, the MRI findings of a duodenal diverticulum with surrounding inflammation strongly suggest that diverticulitis was the underlying cause of her pancreatitis.

Duodenal diverticula are most commonly diagnosed through CT scans. However, as this case illustrates, MRI with MRCP can be invaluable in patients with pancreatitis of unclear etiology, especially when common causes have been excluded. These advanced imaging techniques help rule out de novo stones, malignancy, or other complications. Our patient's case highlights the importance of considering duodenal diverticulitis in the differential diagnosis of idiopathic pancreatitis.

Management of duodenal diverticula depends on the clinical presentation. Asymptomatic cases typically do not require any intervention. For patients presenting with diverticulitis, initial treatment involves broad-spectrum antibiotics. In cases where symptoms persist, endoscopic intervention may be considered. Surgical diverticulectomy is reserved for patients who fail conservative management or develop complications such as perforation [12].

## Conclusions

Duodenal diverticula, while often asymptomatic, represent a significant and frequently overlooked cause of acute pancreatitis, particularly when common etiologies like gallstones or alcohol are absent. This case highlights the critical importance of maintaining a high index of suspicion and utilizing advanced cross-sectional imaging, such as MRCP, to investigate seemingly idiopathic pancreatitis. Failure to identify this underlying cause can lead to recurrent episodes, increased patient morbidity, and misdirected diagnostic and therapeutic efforts. Accurate diagnosis is paramount, as it shifts the management strategy toward targeted conservative care with antibiotics and supportive measures, thereby avoiding unnecessary and potentially harmful invasive procedures like endoscopic retrograde cholangiopancreatography (ERCP).

Ultimately, this case underscores the need for greater clinical awareness and the development of structured diagnostic algorithms for pancreatitis of unclear origin. Establishing interdisciplinary guidelines would aid clinicians in the effective and timely management of complicated duodenal diverticula. Future research should aim to clarify the precise pathophysiological links between diverticulitis and pancreatitis and to prospectively evaluate the long-term outcomes of conservative versus interventional strategies. By integrating advanced imaging into the workup of unexplained pancreatitis, clinicians can enhance diagnostic accuracy, optimize patient care, and prevent the complications associated with this underappreciated condition.
